# Medical Management of Heparin-Induced Thrombocytopenia Causing Acute Adrenal Insufficiency

**DOI:** 10.7759/cureus.13374

**Published:** 2021-02-16

**Authors:** Samridhi Sinha, Wael Kalaji, Mudita Patel, Jad Sargi, Louis Gerolemou

**Affiliations:** 1 Internal Medicine, The Brooklyn Hospital Center, Brooklyn, USA; 2 Pulmonary and Critical Care, The Brooklyn Hospital Center, Brooklyn, USA; 3 Critical Care Medicine, The Brooklyn Hospital Center, Brooklyn, USA

**Keywords:** heparin-induced thrombocytopenia, immune mediated phenomenon, adrenal insufficiency

## Abstract

Heparin-induced thrombocytopenia is an immune-mediated reaction to heparin and heparin analogs, which results in an acquired hypercoagulability syndrome resulting in paradoxical arterial and venous thrombosis leading to thrombocytopenia. Organs with high vascularity, such as the adrenal glands, are at an increased risk of injury in heparin-induced thrombocytopenia due to thrombus formation in the adrenal vein causing adrenal insufficiency. The standard of treatment remains discontinuation of heparin and heparin analogs and starting corticosteroids and non-heparin antithrombotic therapy such as argatroban.

## Introduction

Patients on heparin therapy for either venous thromboembolism treatment or prophylaxis have an increased thrombotic risk due to heparin-induced thrombocytopenia. Heparin-induced thrombocytopenia is seen in patients either on unfractionated heparin or on low molecular weight heparin. It is an immune-mediated reaction to heparin causing a prothrombotic state which causes paradoxical arterial and venous thrombosis leading to thrombocytopenia. Adrenal glands are susceptible to injury as they are highly vascular structures. Acute adrenal insufficiency due to heparin-induced thrombocytopenia should be high on differentials for patients on heparin therapy, if the patient develops pain in the abdomen, hypotension, and fever with thrombocytopenia. We report a case of heparin-induced thrombocytopenia leading to acute adrenal insufficiency in a critically ill patient and was treated with discontinuation of heparin and starting corticosteroids with improvement in the clinical outcome for the patient.

## Case presentation

**Figure 1:**
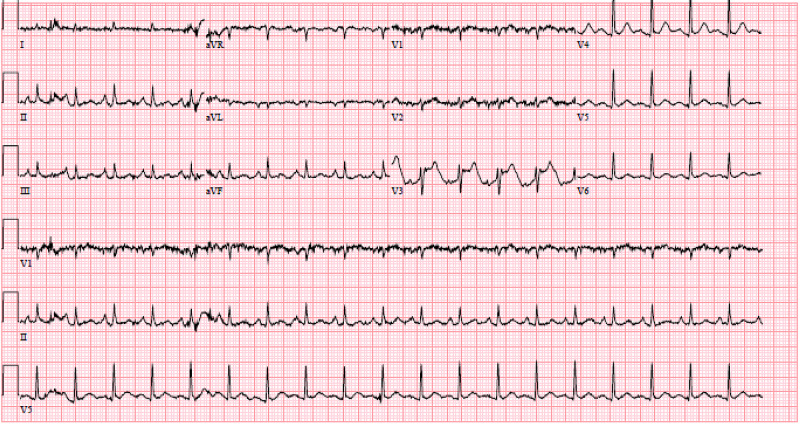
EKG showing non-ST segment elevated myocardial infarction

A 64-year-old female with a past medical history of end-stage chronic obstructive pulmonary disease, active recreational heroin use, and previous deep vein thrombosis of lower extremities was admitted to the medical intensive care unit for acute on chronic hypercarbic respiratory failure secondary to opioid overdose and exacerbation of end-stage chronic obstructive pulmonary disease requiring intubation and mechanical ventilation. At the time of admission, the patient was hypothermic at 93.7 Fahrenheit, tachycardic to 116 bpm with elevated white blood cells (WBC) count, and was started on empiric antibiotics. Blood work showed troponin of 0.8 ng/mL, platelet at 216,000/µL, and hemoglobin at 9.6 g/dL. The patient was also found to have non-ST segment elevated myocardial infarction, at the time of admission, for which the patient was started on heparin drip for anticoagulation (Figure 1).

The patient’s clinical course improved, with normalization of vitals and leukocytosis and the patient was extubated after three days of admission. Four days after the initiation of heparin drip, the patient had an acute drop platelet to 50,000/µL with hemoglobin of 3.3 g/dL. The 4T score was 6 which signified high probability (64%) for heparin-induced thrombocytopenia and heparin drip was discontinued. The patient was also found to be hypotensive with mean arterial pressure ranging between 58-63 mmHg and had a low-grade fever. When asked about pain in the abdomen, the patient complained of diffuse body pain due to opioid withdrawal. The direct Coombs test was negative. Other laboratory values were remarkable for prolonged prothrombin time (PT) and partial thromboplastin time (PTT), elevated d-dimer, low fibrinogen, suggestive of disseminated intravascular coagulopathy, secondary to adrenal insufficiency. The patient also developed microangiopathic hemolytic anemia with low hemoglobin, high lactic dehydrogenase (LDH), low fibrinogen, and low haptoglobin and peripheral blood showing schistocytes secondary to disseminated intravascular coagulopathy.

Computed tomography of the head was negative for hemorrhage and computed tomography abdomen/pelvis, and computed tomography angiogram of abdomen/pelvis were negative for retroperitoneal bleeding. The adrenal gland could not visualized be on a computed tomography angiogram of the abdomen and pelvis.

The patient’s hemodynamic instability was most likely due to adrenal insufficiency. The patient was transfused multiple units of red blood cells and fresh frozen plasma and was started on stress dose steroids with hydrocortisone, ascorbic acid, and thiamine (HAT therapy) after which her blood pressure improved with resolution of thrombocytopenia, and the patient was transferred from medical intensive care unit to the general medical floor. The patient, unfortunately, died due to complications related to the end-stage chronic obstructive pulmonary disease.

## Discussion

Heparin-induced thrombocytopenia occurs in patients who are undergoing heparin treatment, resulting in an acquired hypercoagulability syndrome. It is defined as an immune-mediated reaction to heparin products resulting in a paradoxical arterial and venous thrombosis leading to thrombocytopenia [1].

Heparin-induced thrombocytopenia is an autoimmune phenomenon involving immunoglobulin G (IgG) antibodies that attach to the heparin-platelet factor-4 protein complex leading to platelet activation. The induced platelet activation leads to systemic thrombosis and platelet consumption, which eventually presents as thrombocytopenia. The adrenal glands in particular are at a higher risk of injury due to heparin-induced thrombocytopenia, due to their vascularity. The vascular supply to each adrenal gland is drained by a single central adrenal vein, which is highly susceptible to outflow obstruction. The systemic thrombosis caused by heparin-induced thrombocytopenia can cause adrenal vein thrombosis which subsequently leads to arterial adrenal gland hemorrhage and eventual life-threatening adrenal crisis [2]. The immediate discontinuation of all heparin products is necessary to reverse heparin-induced thrombocytopenia as well as the concurrent use of stress-dose steroids in the case of heparin-induced thrombocytopenia causing adrenal insufficiency [3]. Our patient’s heparin-induced thrombocytopenia resolved with the cessation of heparin products and her adrenal insufficiency responded appropriately to stress-dose steroids.

## Conclusions

Heparin-induced thrombocytopenia, which is an immune-mediated disorder, can cause adrenal insufficiency and should be suspected if patients have abdominal pain and fever with hypotension. Our case underlines the importance of maintaining a high index of awareness for the occurrence and impact of the recognition of heparin-induced adrenal insufficiency and its implications on the clinical course of critically ill patients and treating this subset of patients with discontinuation of heparin and starting corticosteroids.
